# Supplementary material for: NADPH Oxidase Inhibition Promotes Brain Resilience by Attenuating Tauopathy and Neuroinflammation in Alzheimer's Disease
Source: Adv Sci (Weinh). 2025 Aug 18;12(42):e05495. doi: 10.1002/advs.202505495 (PMC12622451; doi:10.1002/advs.202505495)
Supplement: Supplementary file 1 — Supporting Information [file ADVS-12-e05495-s001.docx]

**Supplementary Information**

**NADPH Oxidase Inhibition Promotes Brain Resilience by Attenuating Tauopathy and Neuroinflammation in Alzheimer’s Disease**

Jihyeon Lee^1, 2^, Seunghwan Sim^3^, Yinglan Jin^3^, Eun Young Byeon^4^, Su Jin Kim^4^, Sujin Yun^2^, Hye Eun Lee^1^, Da Un Jeong^1^, Jung Min Suh^2^, In Hye Lee^2^, Ho-Young Lee^4, 5^, Yongseok Choi^3,^*****, and Yun Soo Bae^1, 2,^*****

^1^Celros Biotech, Seoul 03760, Korea.

^2^Department of Life Sciences, Ewha Womans University, Seoul 03760, Korea.

^3^School of Life Sciences and Biotechnology, Korea University, Seoul 02841, Korea.

^4^Department of Nuclear Medicine, Seoul National University Bundang Hospital, Seongnam, Korea.

***Corresponding author:** e-mail: [baeys@ewha.ac.kr](mailto:baeys@ewha.ac.kr); [ychoi@korea.ac.kr](mailto:ychoi@korea.ac.kr)


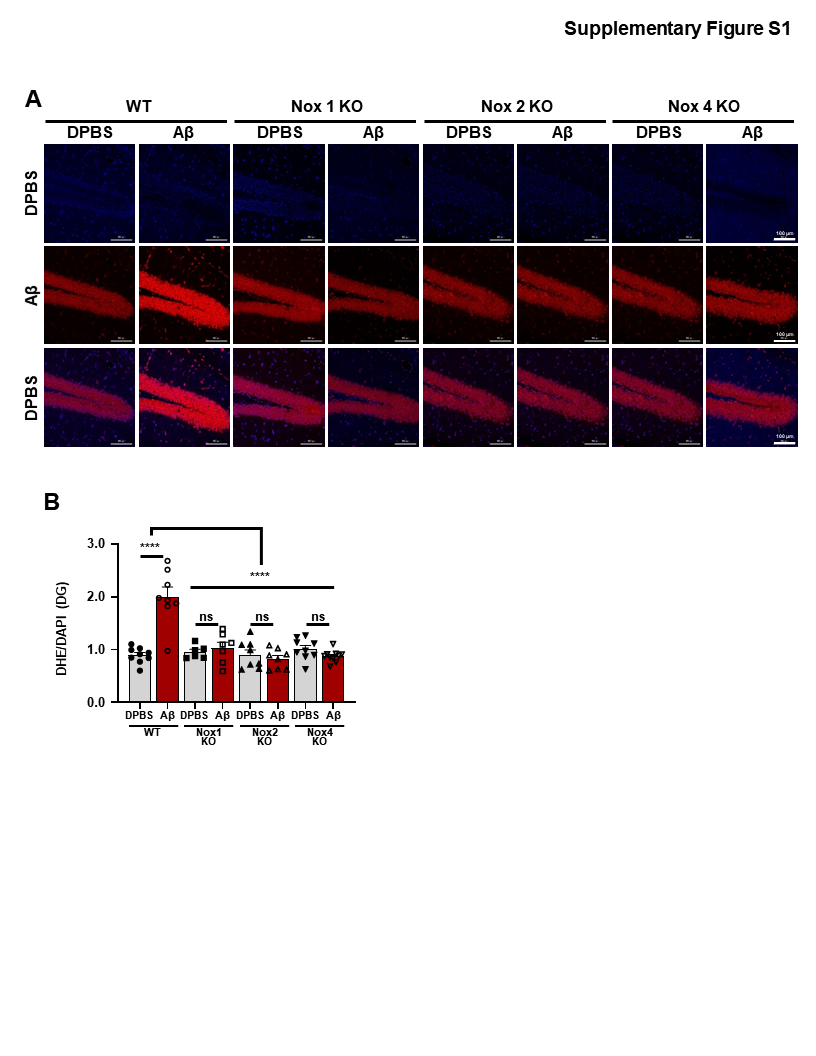


**Supplementary Figure S1.** Deletion of different NADPH oxidase (Nox) isoforms reactive oxygen species (ROS) generation in the hippocampus following amyloid beta (Aβ) injection. Representative images (n = 6–10 per group), Scale bar = 100 µm (A). Quantification of dihydroethidium (DHE) levels (B). Data are expressed as mean ± SEM. One-way ANOVA: F (7, 57) = 18.15, p=0.0010. *p<0.05, **p<0.01, ***p<0.001, ****p<0.0001, as determined by one-way ANOVA followed by Tukey’s post-hoc test.


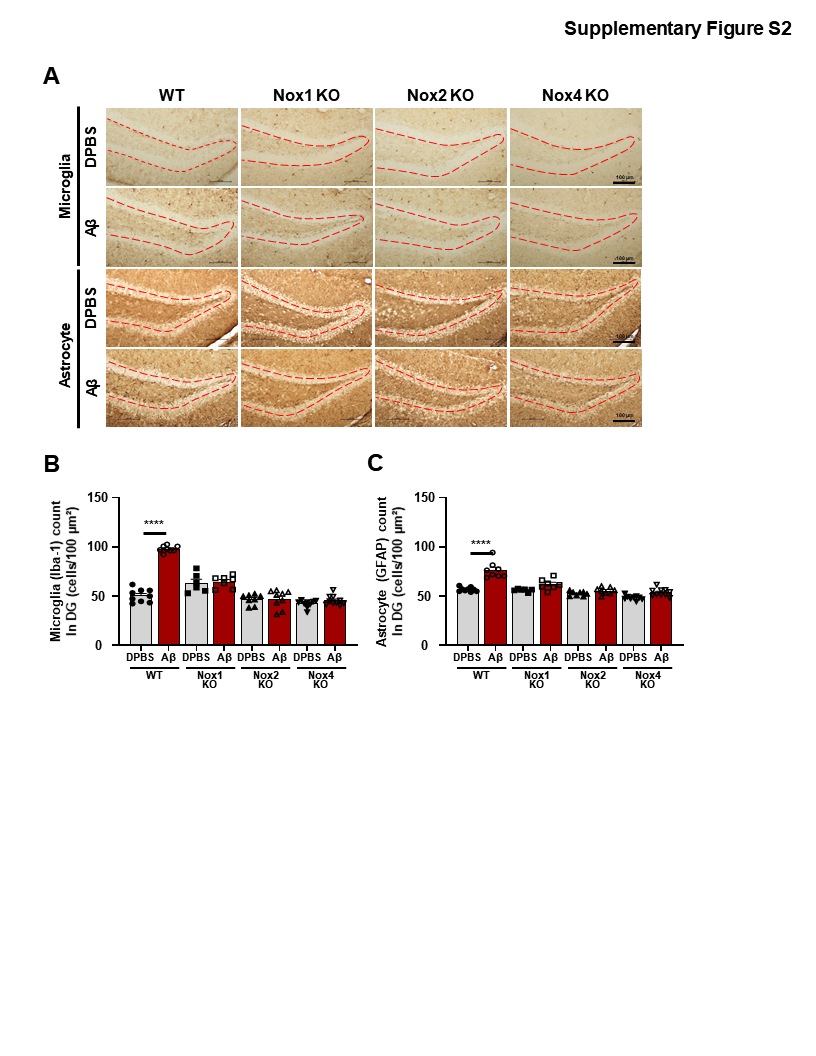


**Supplementary Figure S2.** Deletion of different Nox isoforms modulates microglial and astrocytic activation in the hippocampus following Aβ injection. Representative images of Iba-1 (ionized calcium-binding adapter molecule 1. top two rows) and GFAP (glial fibrillary acidic protein, bottom two rows) immunostaining in the dentate gyrus (DG) of wild-type (WT), Nox1-/-, Nox2-/-, Nox3-/-, and Nox4-/- mice after intracerebroventricular injection of Aβ1-42 (n = 6–10 per group). Scale bar = 100 µm. (A). Immunopositive cells within the red dashed boundaries were counted. In WT mice, Aβ injection markedly increased the number of Iba-1– and GFAP–positive microglia and astrocytes, while Nox-deficient mice showed varying degrees of attenuation. The bar graphs below show quantification of Iba-1(B) (F (7, 58) = 68.93, p < 0.0001)– and GFAP(C)(F (7, 57) = 32.74, p < 0.0001)–positive cell densities in the dentate gyrus. All quantitative data in this figure are shown as mean ± SEM. *p<0.05, **p<0.01, ***p<0.001, ****p<0.0001, as determined by one-way ANOVA followed by Tukey’s post-hoc test.


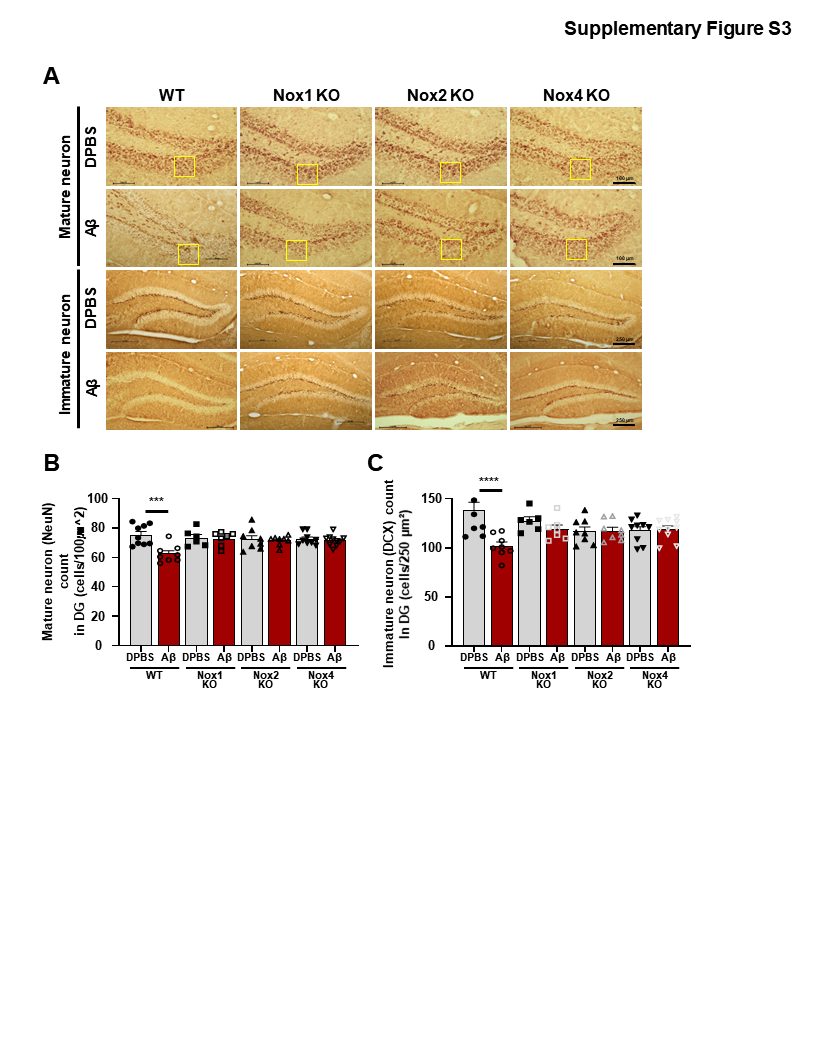


**Supplementary Figure S3.** Nox isoform deficiency attenuates the loss of both mature and immature neurons following Aβ injection. Representative images of NeuN (neuronal nuclei, top two rows, Scale bar = 100 µm) and DCX (doublecortin, bottom two rows, Scale bar = 250 µm) immunostaining in the dentate gyrus of wild-type (WT), Nox1-/-, Nox2-/-, Nox3-/-, and Nox4-/- mice after intracerebroventricular injection of Aβ₁₋₄₂ (n = 6–10 per group)(A). Yellow boxes indicate the regions used for quantification. NeuN staining marks mature neuronal cell in the granule cell layer, while DCX staining reflects the presence of immature neurons. In WT mice, Aβ injection resulted in a reduction of NeuN-positive cells, whereas Nox-deficient mice showed a tendency toward preserved numbers of both mature (NeuN-positive) and immature (DCX-positive) neurons. These findings suggest that deletion of Nox isoforms protects 5xFAD mice from mature neuronal loss and preserves the regenerative pool of immature neurons. Quantification of NeuN (B)(F (7, 57) = 4.124, p=0.0010) and DCX (C)(F(7, 57) = 4.124, p=0.0010) immunoreactivity is shown in the bar graphs below. All quantitative data in this figure are shown as mean ± SEM. *p<0.05, **p<0.01, ***p<0.001, ****p<0.0001, as determined by one-way ANOVA followed by Tukey’s post-hoc test.


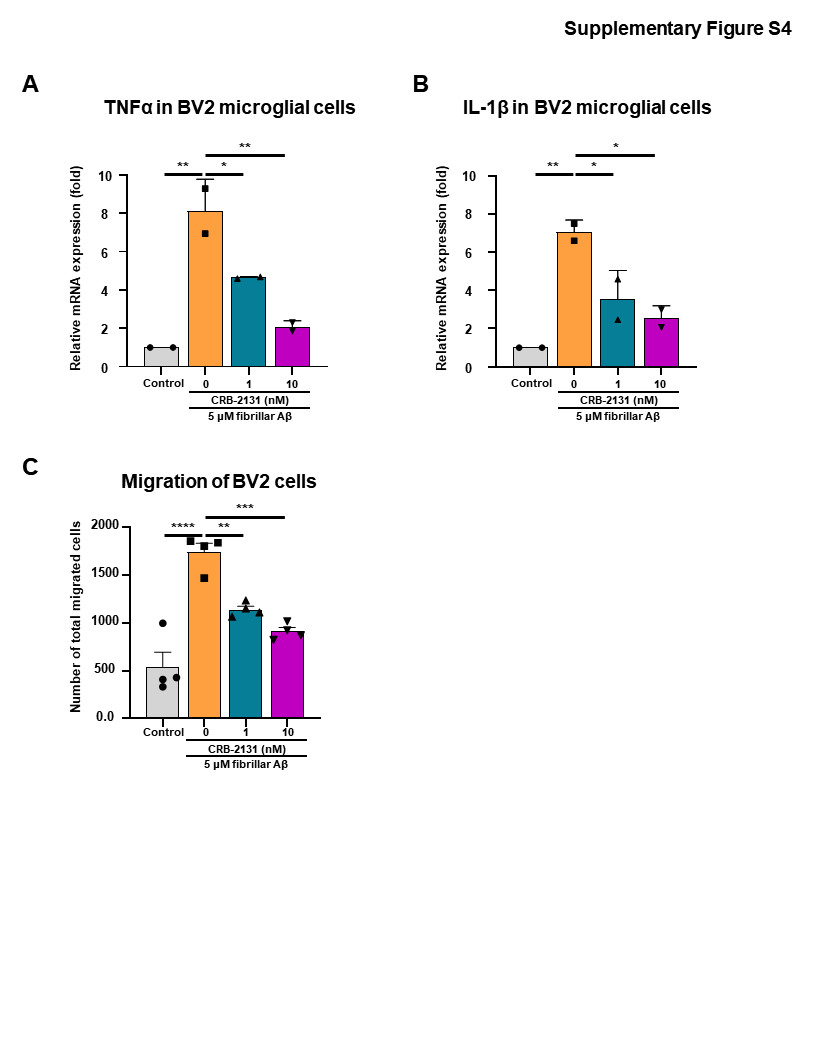


**Supplementary Figure S4.** The mRNA levels of tumor necrosis factor alpha (TNF-α) (A) and interleukin 1 beta (IL-1β) (B) by BV2 microglial cells and BV2 microglial-cell migration (C). Inhibitory activity of CRB-2131 on Aβ-mediated TNFα (F (3, 4) = 27.95, p=0.0038)(A) and IL-1β (F (3, 4) = 16.98, p=0.0097)(B) in BV2 microglial cells. (N=2), Quantification of transwell migration assay (F (3, 12) = 29.09, p<0.0001) (C) (N=4) All quantitative data in this figure are shown as mean ± SD. *p<0.05, **p<0.01, ***p<0.001, ****p<0.0001, as determined by one-way ANOVA followed by Dunnett’s multiple comparisons test.


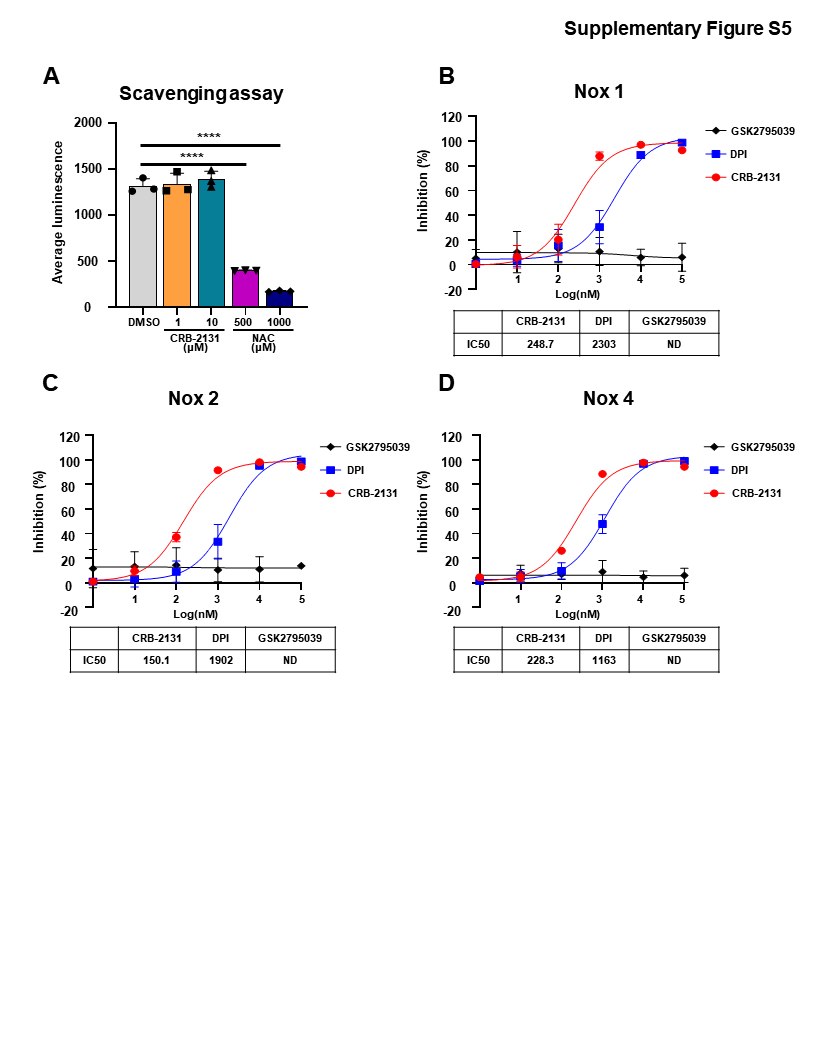


**Supplementary Figure S5.** Evaluation of the direct ROS scavenging activity and Nox isozyme-selective inhibition by CRB-2131. Lucigenin-based chemiluminescence assay evaluating direct ROS scavenging activity. CRB-2131 (1.0 or 10 μM) did not attenuate H₂O₂-induced luminescence, indicating a lack of intrinsic ROS scavenging capacity. In contrast, N-acetyl cysteine (NAC, 1 mM) significantly reduced luminescence intensity (F (4, 10) = 186.4, P<0.0001) (A). Dose-dependent inhibition of ROS production by CRB-2131 in membrane fractions isolated from transgenic Drosophila expressing human Nox1 (B), Nox2 (C), or Nox4 (D). ROS generation was measured via lucigenin-based chemiluminescence in the presence of NADPH (500 μM). CRB-2131 reduced Nox-dependent ROS production in a concentration-dependent manner, with lower half-maximal inhibitory concentration (IC₅₀) values compared to DPI and no inhibition observed with GSK2795039. DPI, apocynin, and GSK2795039 were used as positive controls for comparison with established Nox inhibitors. IC₅₀ values were calculated by nonlinear regression analysis (log[inhibitor] vs. response, three-parameter fit). CRB-2131 showed the strongest inhibitory activity (IC₅₀ = 248.7 μM, 95% confidence interval (CI): 177.0–350.4 μM), with a high goodness of fit (coefficient of determination (R²) = 0.965), compared to DPI (IC₅₀ = 2303 μM, R² = 0.958) and GSK2795039, which showed minimal activity (IC₅₀ = 5748 μM, R² = 0.024). Data are presented as mean ± SD. Statistical significance was determined using one-way ANOVA followed by Dunnett’s multiple comparisons test. *p < 0.05, **p < 0.01, ***p < 0.001, ****p < 0.0001.


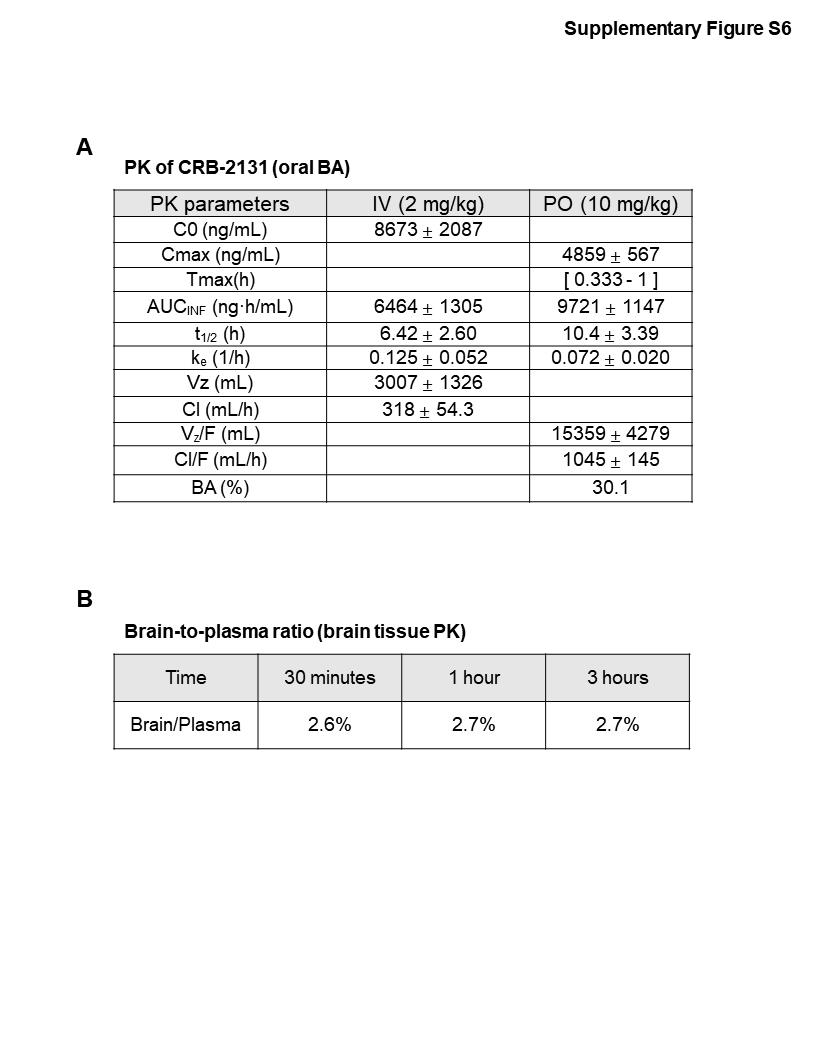


**Supplementary Figure S6.** The pharmacokinetic profile (A) and brain-to-plasma ratio (B) of CRB-2131.


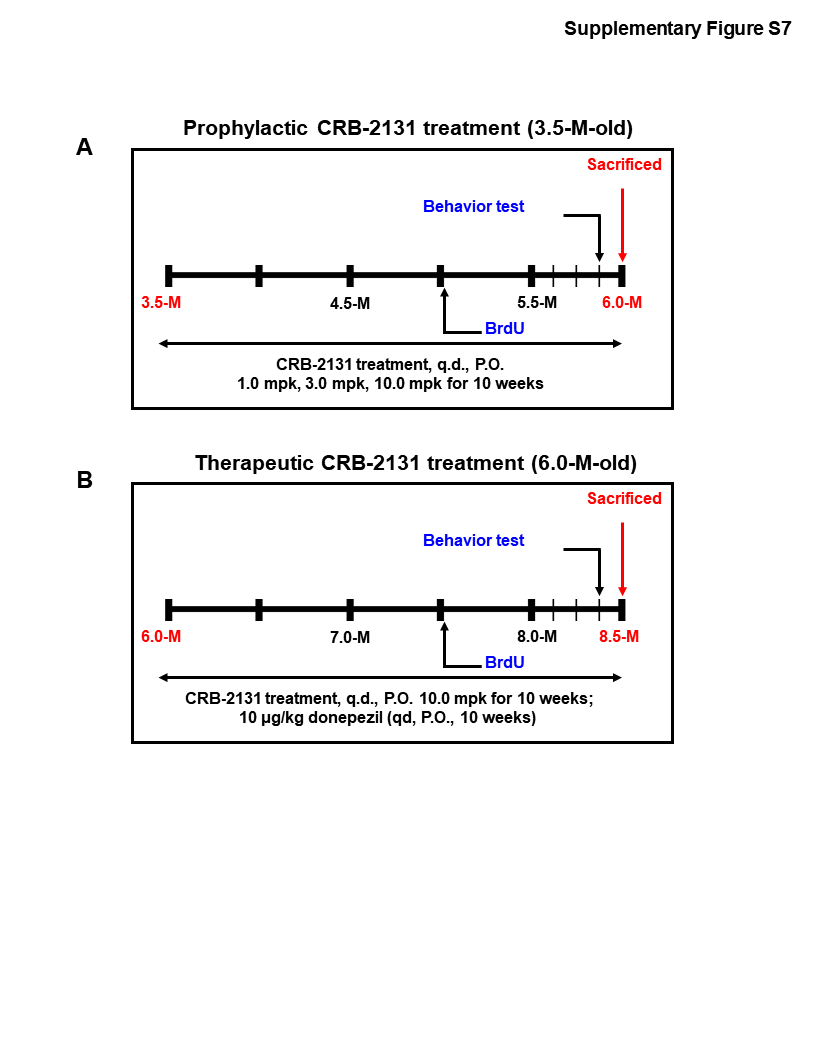


**Supplementary Figure S7.** Schematic depiction of the prophylactic treatment of 3.5-month-old (A) and the therapeutic treatment of 6.0-month-old (B) 5XFAD mice with CRB-2131. P.O., per oral; mpk, mg per kilogram.


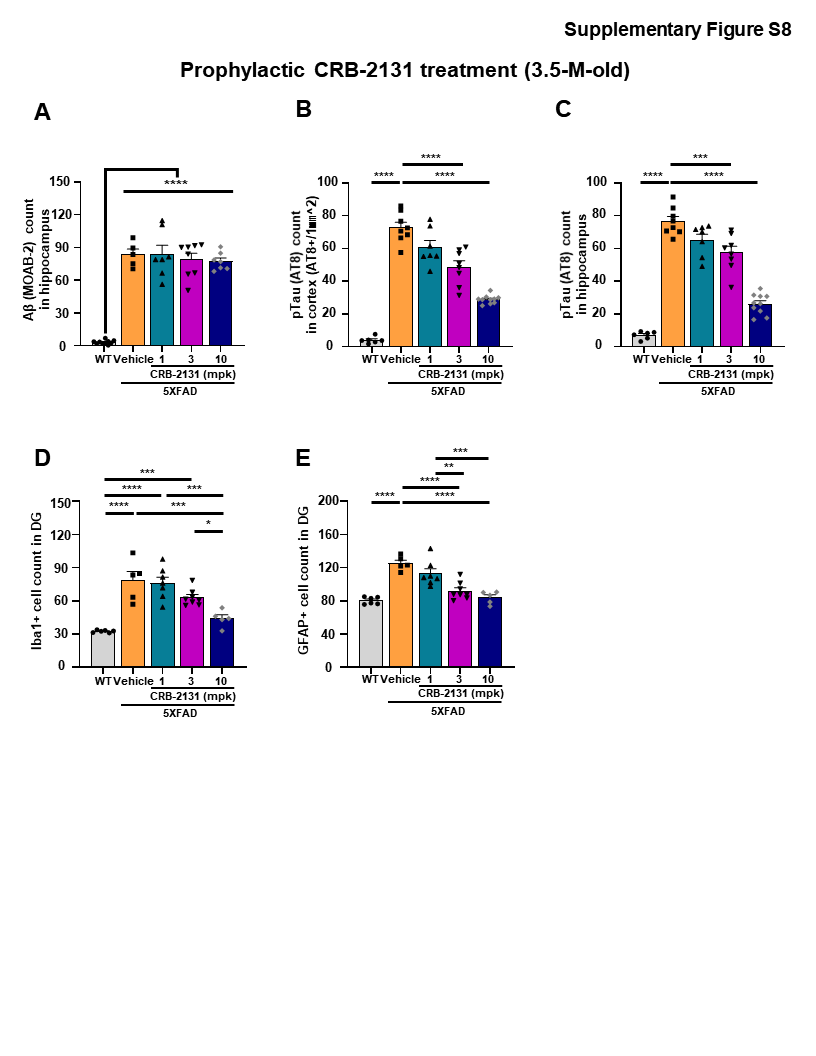


**Supplementary Figure S8.** Quantification of immunohistochemistry of aggregated Aβ in the hippocampus (A) (One-way ANOVA: F (4, 32) = 63.89, p<0.0001). (n = WT (n=10), 5xFAD + vehicle (n=5), CRB-2131 1mpk (n=7), 3mpk (n=8), 10mpk (n=7)), pTau in the cortex (B) (One-way ANOVA: F (4, 34) = 77.93, p<0.0001) and the hippocampus (C) (One-way ANOVA: F (4, 34) = 87.40, p<0.0001), (n = WT (n=6), 5xFAD + vehicle (n=8), CRB-2131 1mpk (n=7), 3mpk (n=8), 10mpk (n=10)), and microglia (Iba1+) (D) (One-way ANOVA: F (4, 26) = 18.72, p<0.0001) and astrocyte (GFAP+) (E) (One-way ANOVA F (4, 26) = 19.51, p<0.0001), (n = WT (n=6), 5xFAD + vehicle (n=5), CRB-2131 1mpk (n=7), 3mpk (n=8), 10mpk (n=5)) in the DG of the hippocampus in 5XFAD mice (3.5-month-old) administrated with prophylactic CRB-2131 treatment. All quantitative data in this figure are shown as mean ± SEM. *p<0.05, **p<0.01, ***p<0.001, ****p<0.0001, as determined by one-way ANOVA followed by Tukey’s post-hoc test.


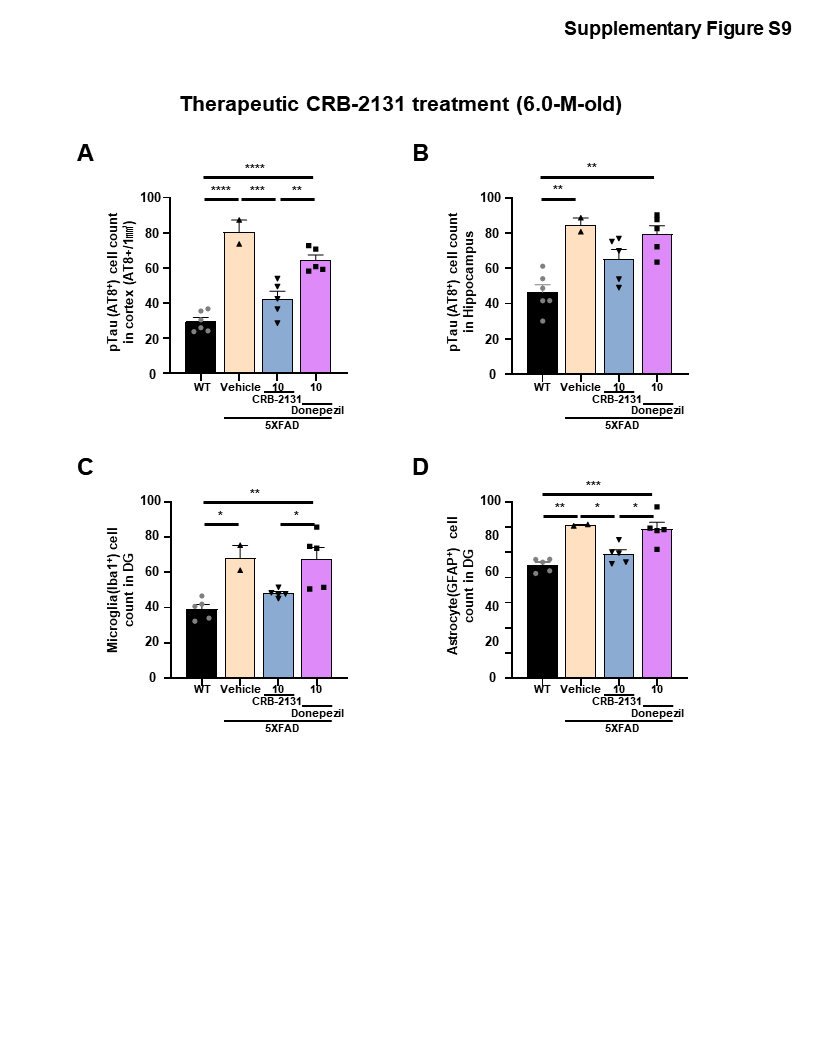


**Supplementary Figure S9.** Quantification of immunohistochemistry of pTau in the cortex (A) (One-way ANOVA: F (3, 14) = 30.91, p<0.0001) and the hippocampus (B) (One-way ANOVA: F (3, 14) = 10.19, p = 0.0008), and microglia (Iba1+) (C) (One-way ANOVA: F (3, 13) = 9.012, p = 0.0017) and astrocyte (GFAP+) (D) (One-way ANOVA: F (3, 13) = 13.15, p = 0.0003) in the DG of the hippocampus in 5XFAD mice (6.0-month-old) administrated with therapeutic CRB-2131 treatment. (n = WT (n=5-6), 5xFAD + vehicle (n=2), CRB-2131 10mpk (n=5), donepezil 10mpk (n=5)). All quantitative data in this figure are shown as mean ± SEM. *p<0.05, **p<0.01, ***p<0.001, ****p<0.0001, as determined by one-way ANOVA followed by Tukey’s post-hoc test.


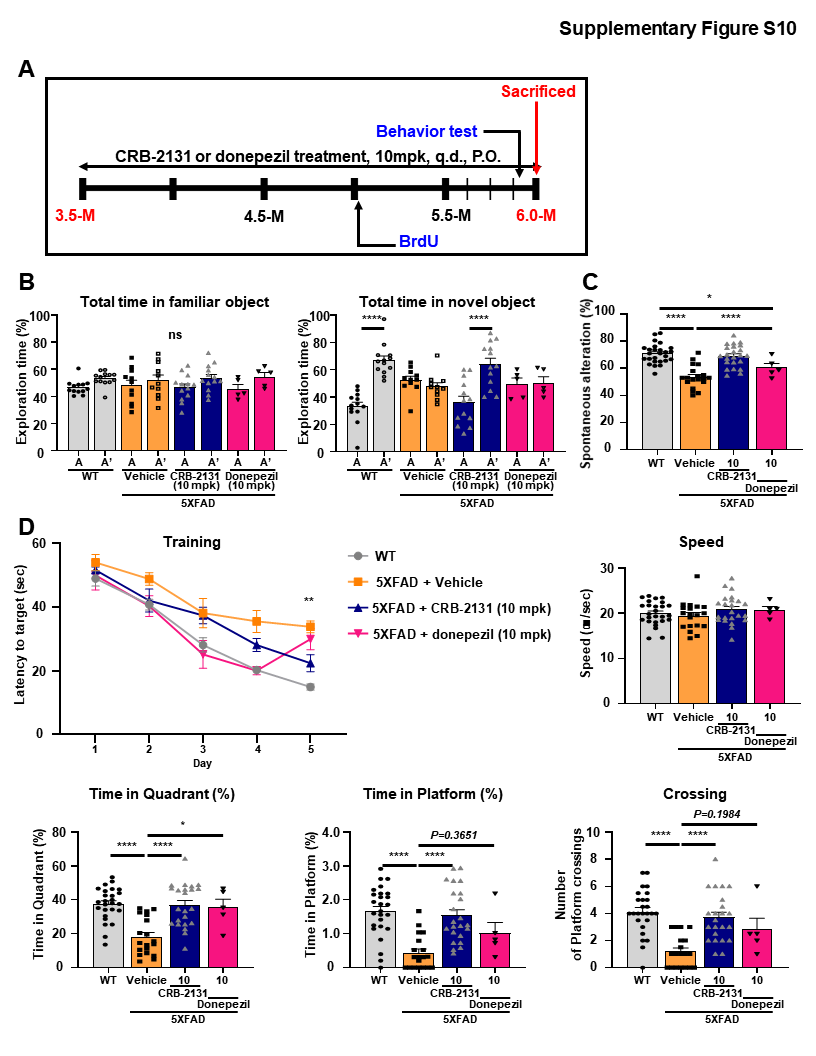


**Supplementary Figure S10.** Comparison of the activity of CRB-2131 and donepezil in behavioral tests (novel object recognition (NOR), Y-maze, and Morris water maze (MWM)) in 5XFAD mice (3.5-month-old). We treated 3.5-month-old 5XFAD mice with 10 weeks of 10 mg/kg CRB-2131 (qd, P.O., 10 weeks) or 10 μg/kg donepezil (qd, P.O., 10 weeks) (A) and then subjected to the NOR (B). Data are expressed as mean ± SEM. One-way ANOVA: F (7, 76) = 11.20, p<0.0001. (n = WT (n=13), 5xFAD + vehicle (n=11), CRB-2131 10mpk (n=13), donepezil 10mpk (n=5)), Y-maze (C) Data are expressed as mean ± SEM. One-way ANOVA: F (3, 69) = 21.57, p<0.0001. (n = WT (n=26), 5xFAD + vehicle (n=19), CRB-2131 10mpk (n=23), donepezil 10mpk (n=5)), and MWM tests (D) The mice underwent 5 consecutive days of training (4 trials per day) with a fixed platform. Data represent mean ± SEM. Two-way repeated measures ANOVA: group effect, F (3, 12) = 9.823, p = 0.0015; day effect, F (2.290, 27.48) = 76.11, p < 0.0001; drug effect, F (12, 48) = 1.868, p = 0.0632, WT vs. 5xFAD+Vehicle on day 5: p = 0.0019. The platform was removed on the sixth day, and the following were measured: the average swimming speed of the mice (D) (F (3, 69) = 0.9141, p=0.4388), the time spent in the platform quadrant (E, left) (F (3, 69) = 12.93, p < 0.0001), the time spent at the place the platform used to be (E, middle) (F(4, 62) = 10.45, p < 0.0001), and the number of times the mice swam over the place the platform used to be (E, right) (F (3, 69) = 13.62, p < 0.0001), (n = WT (n=26), 5xFAD + vehicle (n=19), CRB-2131 10mpk (n=23), donepezil 10mpk (n=5)). All quantitative data in this figure are shown as mean ± SEM. *p<0.05, **p<0.01, ***p<0.001, ****p<0.0001, as determined by one-way ANOVA followed by Tukey’s post-hoc test.


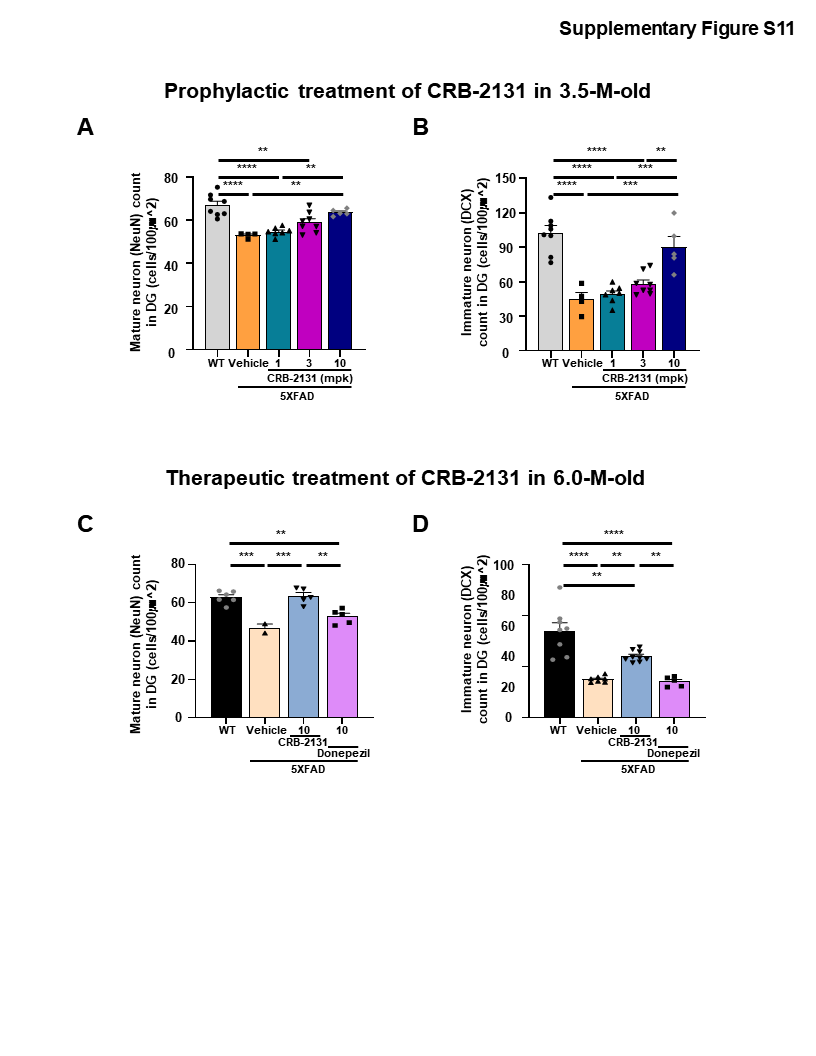


**Supplementary Figure S11.** Quantification of the mature neurons (NeuN+) (A) (F (4, 27) = 15.48, p < 0.0001) and immature neuron (DCX+) (B) (F (4, 27) = 21.64, p < 0.0001) in the DG from 5XFAD mice with prophylactic CRB-2131 treatment (n = WT (n=8), 5xFAD + vehicle (n=4), CRB-2131 1mpk (n=7), 3mpk (n=8), 10mpk (n=5)). Quantification of the mature neurons (NeuN+) (C) (F (3, 14) = 17.16, p < 0.0001) (n = WT (n=6), 5xFAD + vehicle (n=2), CRB-2131 10mpk (n=5), donepezil 10mpk (n=5)) and immature neuron (DCX+) (D) (F (3, 25) = 23.36, p < 0.0001) (n = WT (n=8), 5xFAD + vehicle (n=7), CRB-2131 10mpk (n=9), donepezil 10mpk (n=5)) in the DG from 5XFAD mice with therapeutic CRB-2131 treatment. All quantitative data in this figure are shown as mean ± SEM. *p<0.05, **p<0.01, ***p<0.001, ****p<0.0001, as determined by one-way ANOVA followed by Tukey’s post-hoc test.


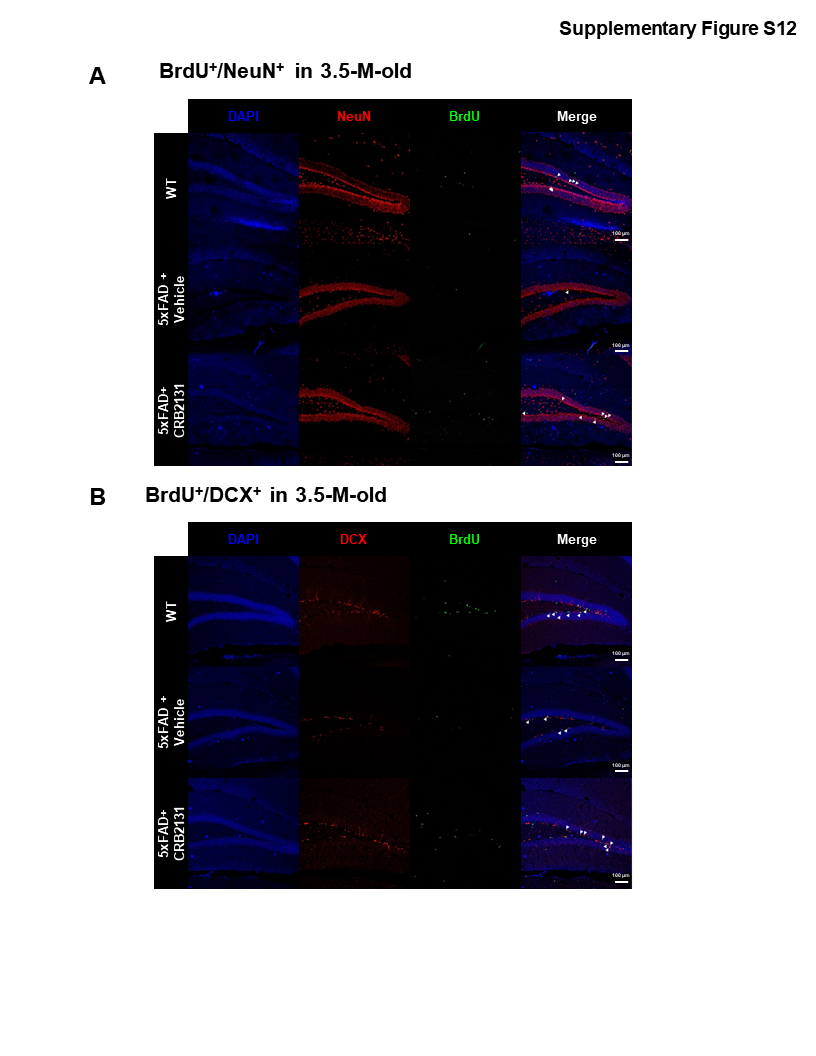


**Supplementary Figure S12.** Representative colocalization images of BrdU with mature neuron (NeuN) (A) and immature neuron (DCX^+^) (B) in DG of 5XFAD mice (3.5-month-old mice, qd, P.O., 10 weeks) with or without CRB-2131 administration (10 mg/kg), scale bar=100μm.


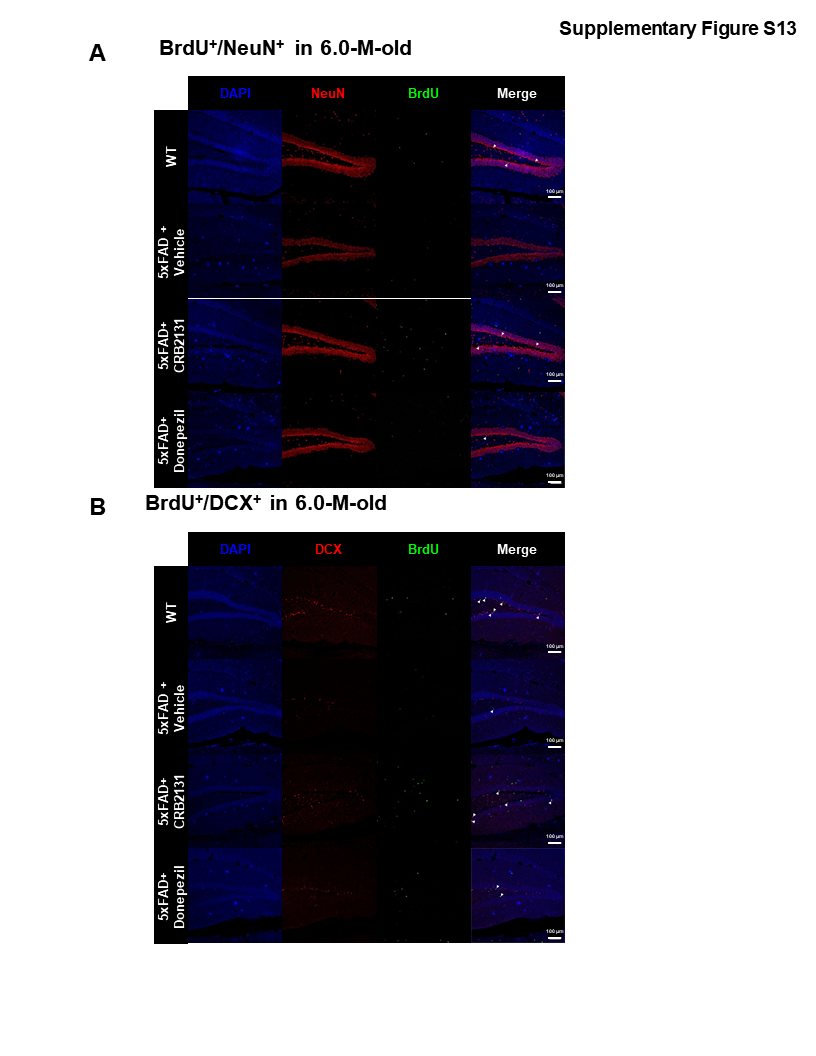


Supplementary Figure S13. Representative colocalization images of BrdU with mature neuron (NeuN) (A) and immature neuron (DCX+) (B) in DG of 5XFAD mice (6.0-month-old mice, qd, P.O., 10 weeks) with or without CRB-2131 administration (10 mg/kg), scale bar=100μm.


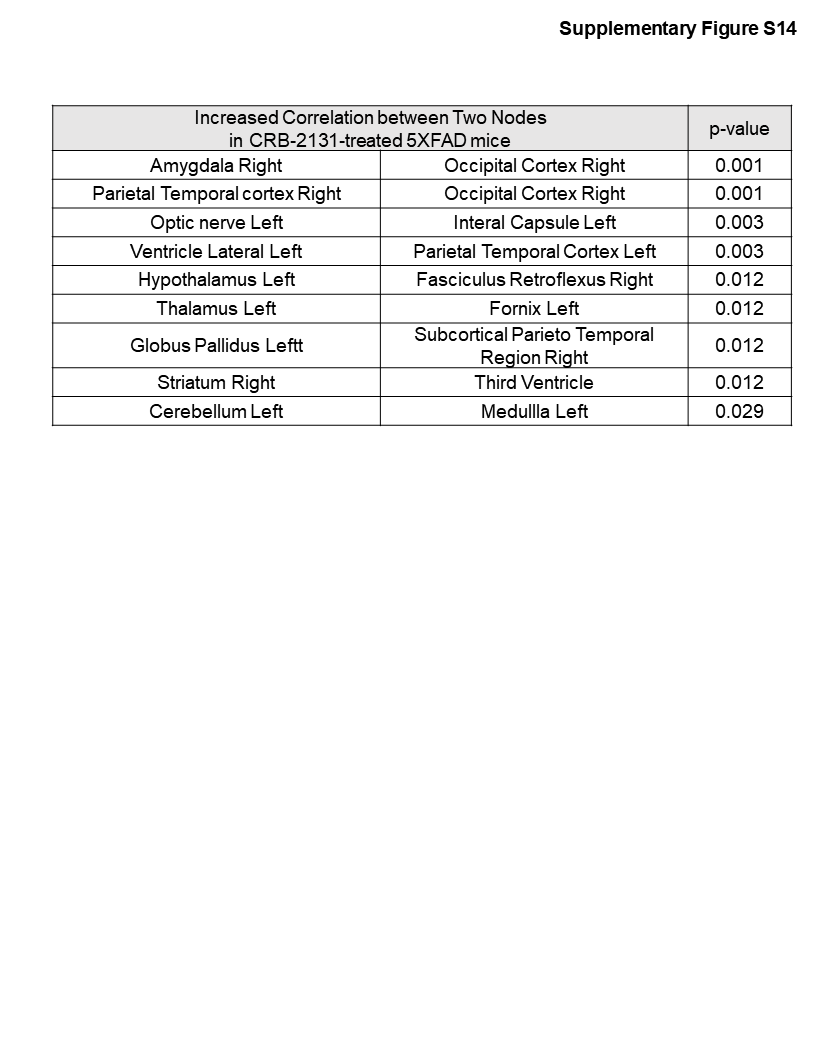


**Supplementary Figure S14.** Prophylactic CRB-2131-treated 5XFAD mice (3.5-month-old mice, *q.d.*, *P.O.*, and 10 weeks) underwent positron emission tomography/computed tomography (PET/CT) at 6 months of age. Altered connectivity between two brain nodes in CRB-2131-treated 5XFAD mice.
